# Efficient electroporation of neuronal cells using synthetic oligonucleotides: identifying duplex RNA and antisense oligonucleotide activators of human frataxin expression

**DOI:** 10.1261/rna.071290.119

**Published:** 2019-09

**Authors:** Xiulong Shen, Sharon Beasley, Jennifer N. Putman, Yanjie Li, Thahza P. Prakash, Frank Rigo, Marek Napierala, David R. Corey

**Affiliations:** 1 Departments of Pharmacology and Biochemistry, UT Southwestern Medical Center at Dallas, Dallas, Texas 75390, USA; 2 MaxCyte Inc., Gaithersburg, Maryland 20878, USA; 3 Department of Biochemistry and Molecular Genetics, UAB Stem Cell Institute, University of Alabama, Birmingham, Alabama 35294, USA; 4 Ionis Pharmaceuticals, Carlsbad, California 92010, USA

**Keywords:** antisense oligonucleotide, electroporation, frataxin, gene activation, therapeutic development

## Abstract

Oligonucleotide drugs are experiencing greater success in the clinic, encouraging the initiation of new projects. Resources are insufficient to develop every potentially important project, and persuasive experimental data using cell lines close to disease target tissue is needed to prioritize candidates. Friedreich's ataxia (FRDA) is a devastating and currently incurable disease caused by insufficient expression of the enzyme frataxin (FXN). We have previously shown that synthetic nucleic acids can activate *FXN* expression in human patient-derived fibroblast cells. We chose to further test these compounds in induced pluripotent stem cell-derived neuronal progenitor cells (iPSC-NPCs). Here we describe methods to deliver oligonucleotides and duplex RNAs into iPSC-NPCs using electroporation. Activation of *FXN* expression is potent, easily reproducible, and potencies parallel those determined using patient-derived fibroblast cells. A duplex RNA and several antisense oligonucleotides (ASOs) with different combinations of 2′-methoxyethyl (2′-MOE), 2′-fluoro (2′-F), and constrained ethyl (cEt) were active, providing multiple starting points for further development and highlighting improved potency as an important goal for preclinical development. Our data support the conclusion that ASO-mediated activation of *FXN* is a feasible approach for treating FRDA and that electroporation is a robust method for introducing ASOs to modulate gene expressions in neuronal cells.

## INTRODUCTION

For almost three decades, oligonucleotide therapeutics made slow progress (Shen and Corey 2018). While a handful of compounds were approved for clinical use, none demonstrated long term success or had a substantial impact on patient care. Within the past several years, however, new drugs have been approved that are making a significant impact in the treatment of heretofore difficult or impossible to treat diseases.

Successful drugs include antisense oligonucleotides (ASOs) Exondys51 (Sarepta, Duchenne muscular dystrophy) (Aartsma-Rus and Krieg 2017), Spinraza (Biogen/Ionis, spinal muscular atrophy) (Corey 2017), Tegsedi (Akcea/Ionis, hereditary transthyretin-mediated amyloidosis) (Keam 2018), and one duplex RNA, Onpattro (Alnylam, hereditary transthyretin-mediated amyloidosis) (Hoy 2018). These compounds have proven that synthetic oligonucleotides can benefit patients and will encourage wider application of oligonucleotide medicine to drug development. One consequence of this success is that more candidates are being considered and more data is needed to justify prioritizing a candidate for clinical development. Criteria for prioritization include unmet need, existence of competing development strategies (small molecule, antibody, gene therapy, etc.), and potential for achieving adequate activity in vivo.

Friedreich's ataxia (FRDA) is a devastating neurological disorder caused by a GAA trinucleotide repeat expansion within intron 1 of the frataxin (FXN) gene (Pandolfo 2009; Bürk 2017). The mutation does not change the composition of FXN protein. Instead, the mutation within the intron reduces *FXN* transcription, RNA, and protein levels. The reduction is only approximately threefold, but it is enough to cause disease. The leading hypothesis explaining reduced FXN protein levels is that the expanded intron binds to the *FXN* chromosomal DNA to form an R-loop that acts as a “brake” to reduce transcription and increase epigenetic silencing markers (Groh et al. 2014a,b; Gerhardt et al. 2016).

Currently, there are no curative treatments and the unmet need for patients is high (Indelicato and Bösch 2018). Because FXN is an intracellular protein that is down-regulated, FRDA is not likely to be a good candidate for curative antibody therapeutics. While small molecules have been reported to up-regulate FXN expression (Sandi et al. 2011; Gottesfeld et al. 2013; Sahdeo et al. 2014; Soragni et al. 2014; Erwin et al. 2017), achieving potent activation in combination with adequate gene specificity is likely to be difficult.

Gene therapy to replace FXN protein expression has met with striking success in mice (Perdomini et al. 2014; Ouellet et al. 2017; Piguet et al. 2018) and holds great promise as a human treatment. Gene therapy, however, continues to confront general challenges and its near term success as a therapy for FRDA remains uncertain (Deverman et al. 2018; Zhang et al. 2018a). Taken together, the status of other therapeutic modalities suggests a continued need for the development of oligonucleotide therapeutics.

We demonstrated previously that duplex RNAs, single-stranded silencing RNAs (ss-siRNAs), and ASOs can target the expanded GAA repeat, reverse R-loop formation, and cause threefold restoration of FXN protein expression (Li et al. 2016, 2018; Shen et al. 2018). These experiments were performed in patient-derived fibroblast cells. Fibroblast cells have several strengths as an experimental system including: (i) The expansion occurs within the endogenous gene, (ii) expression is controlled by natural regulatory mechanisms, and (iii) cell lines derived from several different patients with varied repeat lengths are available, allowing conclusions to be generalized to the overall patient population.

FRDA, however, is not a disease of fibroblast cells. Moreover, the R-loop mechanism is unusual—much different from the standard mechanisms of gapmer ASOs that target mRNA that lead to degradation or steric block ASOs that target pre-mRNA to affect gene splicing. These facts create uncertainty—it was not clear that the activation of gene expression we observed in fibroblast cells will also characterize more disease-relevant cell types. This uncertainty is an important obstacle to efforts aimed at preclinical development.

To further test the hypothesis that nucleic acid activators of *FXN* expression might be candidates for drug development and help justify investment in animal trials, we chose to test activation in induced pluripotent stem cell-derived neuronal progenitor cells (iPSC-NPCs). However, before we could test iPSC-NPCs it was essential that we develop efficient methods for introducing nucleic acids into them.

In this paper, we first describe the development of rapid and robust electroporation protocols for the efficient introduction of gene silencing nucleic acids into iPSC-NPCs. These protocols were proven to be simple and easily reproducible. We then demonstrate that elevated *FXN* RNA and protein levels can be achieved and evaluate compound potencies, moving oligonucleotide activators of *FXN* expression one step closer as competitive candidates for drug development.

## RESULTS

### Experimental design

**FIGURE 1.**
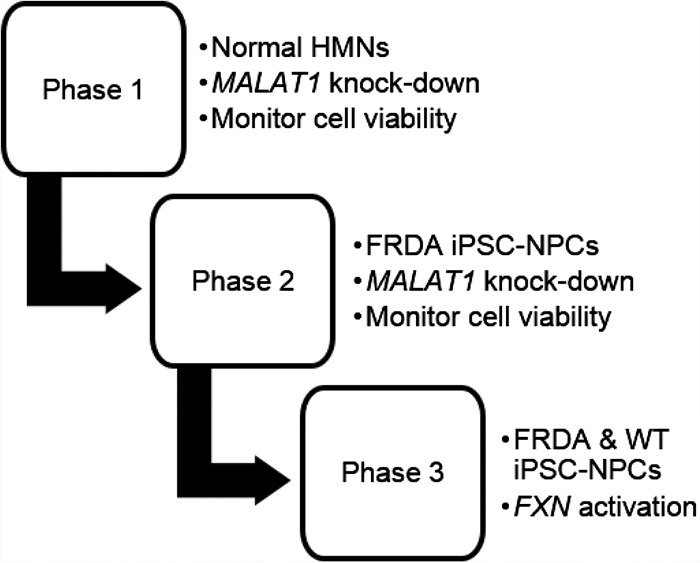
Experimental design. Phase 1: establish protocol with benchmark gene (*MALAT1*) and commercial neuronal cell line. Phase 2: establish protocol using FRCD patient-derived iPSC-NPCs. Phase 3: test activation of *FXN* expression. (HMNs) Human motor neurons, (FRDA) Friedreich's ataxia, (NPCs) neuronal progenitor cells, (WT) wild-type.

Our goals were to develop an efficient method for introducing synthetic nucleic acids into neuronal cells and then test anti-GAA nucleic acids that target the intronic repeat region for their ability to activate *FXN* expression (Fig. 1). To introduce nucleic acids into cells we chose the MaxCyte transfection system (Fratantoni et al. 2003) because preliminary data suggested that it combined high transfection efficiency, robust modulation on gene expression, and low toxicity.

The MaxCyte system is designed for clinical use and improves primary cell transfection viability by using inert metals instead of aluminum in the electroporation electrodes to avoid toxic metal ions leaching into the cell suspension. The MaxCyte system also has preset protocols for most cell types. While the MaxCyte system is commonly used for engineering of multiple primary cell types by transfecting plasmid DNA, mRNA, and RNPs (De Ravin et al. 2016, 2017; Hung et al. 2018), there were no published reports using ASOs or dsRNAs and our first goal was to identify conditions that combined potency with low toxicity.

**FIGURE 2.**
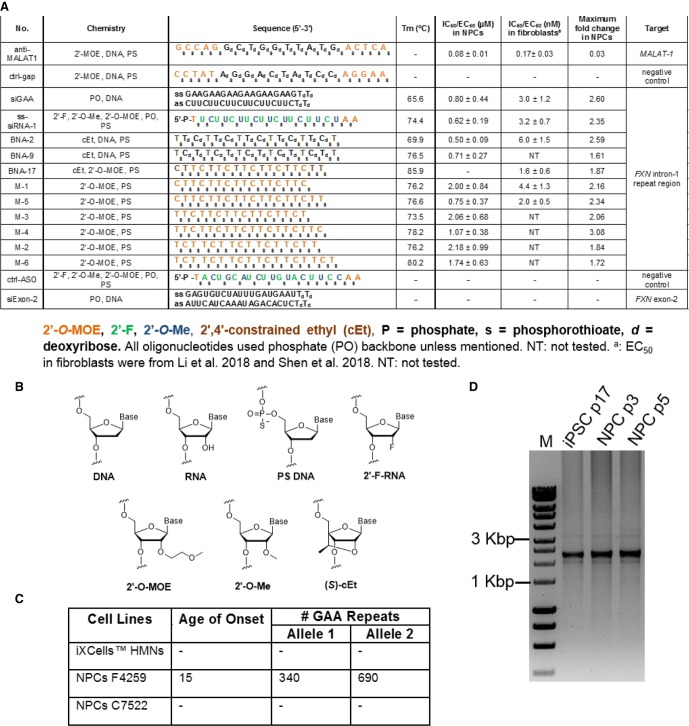
Synthetic nucleic acids and cell lines used in this study. (*A*) Duplex RNA and single-stranded oligonucleotides. (*B*) Structure of chemical modifications. (*C*) Cell lines used in this study. (*D*) PCR analysis of the number of GAA repeats in F4259 iPSCs and NPCs. Analyses were conducted as described in Li et al. (2015). Somatic instability of the long repeats can be detected. p designates passage number. (HMNs) Human motor neurons, (NPCs) neuronal progenitor cells, (iPSC) induced pluripotent stem cells.

We first tested electroporation protocols for neuronal cells to identify the conditions combining high efficiency and low toxicity (Fig. 1). Like the target *FXN* intronic RNA, *MALAT-1* is a nuclear RNA. We targeted MALAT-1 with a complementary ASO gapmer consisting of a central DNA “gap” designed to recruit RNase H upon hybridization to RNA and flanking 2′-methoxyethyl (2′-MOE) regions to enhance binding (Fig. 2A,B). The anti-*MALAT1* ASO gapmer was chosen because it was a benchmark compound well known for its ability to recognize and silence a nuclear RNA target with good potency.

We used commercially obtained neuronal cells (iPSC-derived HMN, healthy donor) for this initial testing. We performed a second round of optimization using the anti-*MALAT1* gapmer and iPSC-derived neuronal progenitor cells (iPSC-NPCs) F4259 that originated from an FRDA patient and contained 340 and 690 expanded GAA repeat within the *FXN* gene (Fig. 2C,D). The final stage was testing synthetic compounds with various modifications (Fig. 2A,B) complementary to the expanded GAA repeat for their ability to activate FXN expression in iPSC-NPCs.

### Electroporation protocols for neuronal cells

**FIGURE 3.**
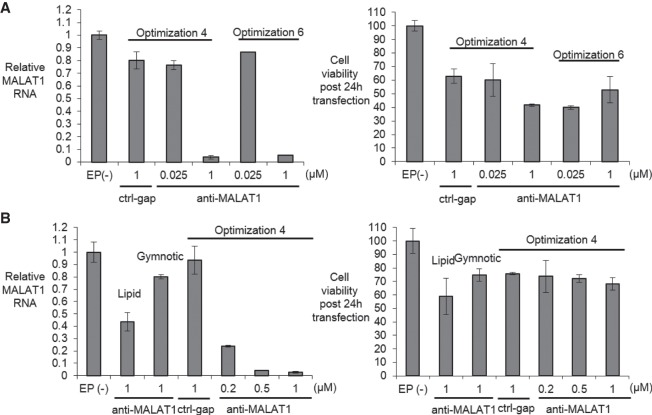
Identifying an electroporation protocol using iXCells human motor neurons (HMNs). (*A, left*) Relative *MALAT-1* RNA levels measured by q RT-PCR when transfected with control gapmer ctrl-gap (1 µM) and anti-MALAT1 gapmer anti-MALAT1 using Optimization 4 and 6 (*n* = 2) post 24 h transfection. (*Right*) Cell viability post 24 h transfection measured by trypan blue staining (*n* = 2) using 1× trypsin as detachment reagent. (*B, left*) Relative *MALAT1* RNA levels measured by q RT-PCR when transfected with control gapmer ctrl-gap (1 µM) and anti-MALAT1 gapmer anti-MALAT1 using Optimization 4 (*n* = 2) compared with Lipofectamine stem transfection reagent or gymnotic delivery post 24 h transfection. (*Right*) Cell viability post 24 h transfection measured by trypan blue staining (*n* = 2) using accutase + 10 µM Y27632. EP(−) is non-oligo treatment, no electroporation control. All data are presented as ±STDEV.

We first tested the knockdown of *MALAT1* RNA in wild-type HMNs using qRT-PCR to evaluate efficiency. Both pulse programs tested yielded efficient knockdown of *MALAT1* expression when ASO anti-MALAT1 was present at 1 µM (Fig. 3A) but also a >40% percent reduction in cell viability, suggesting the need to modify our protocol. To improve viability, we used accutase rather than trypsin to dissociate the cells from the plastic cell culture plates. We also used a rho-associated kinase (ROCK) inhibitor Y27632 that can reduce dissociation-induced apoptosis (anoikis) with accutase (Watanabe et al. 2007; Koyanagi et al. 2008).

Using this modified protocol, we observed >95% knockdown of *MALAT1* combined with >70% viability after electroporation (Fig. 3B). Noncomplementary gapmer ctrl-gap did not knock down *MALAT1* expression. We then compared the transfection efficiency of electroporation with Lipofectamine stem transfection reagent and gymnotic free uptake mediated transfection. Optimization 4 was used because it is lower energy and gentler to the cells than Optimization 6.

When gapmer anti-MALAT1 was delivered by lipid transfection or gymnotic delivery we observed a 60% and 20% reduction of *MALAT1* RNA, respectively. Both values are much less potent than electroporation (80%–95%). These results established the feasibility of using the MaxCyte system for electroporation of ASOs into human neuronal cells and that electroporation could be more effective than by other methods.

**FIGURE 4.**
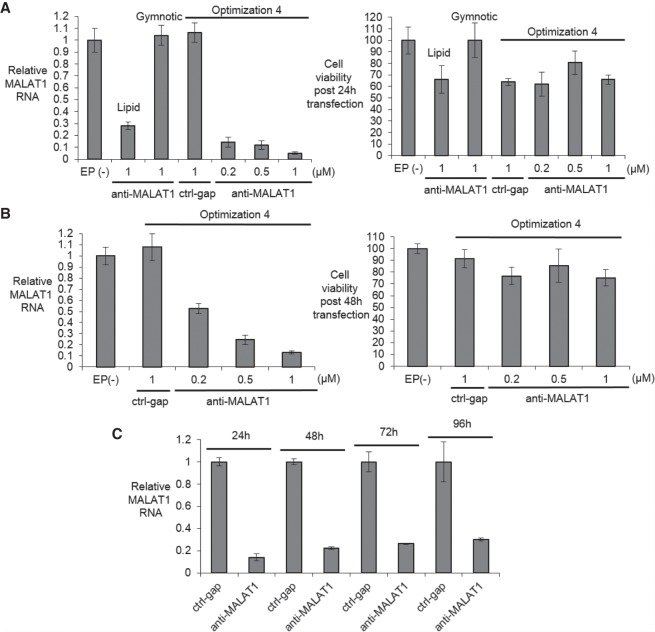
Identifying an electroporation protocol for FRDA patient-derived F4259 iPSC-NPCs. (*A, left*) Relative *MALAT1* RNA levels measured by RT-qPCR when transfected with control gapmer ctrl-gap and anti-MALAT1 gapmer anti-MALAT1 using Optimization 4 (*n* = 2) compared with Lipofectamine stem transfection reagent and gymnotic delivery post 24 h transfection. (*Right*) Cell viability post 24 h transfection measured by trypan blue staining (*n* = 2). (*B, left*) Relative *MALAT1* RNA levels measured by qRT-PCR when transfected with control gapmer ctrl-gap and anti-MALAT1 gapmer anti-MALAT1 using Optimization 4 (*n* = 4) post 48 h transfection. (*Right*) Cell viability post 48 h transfection measured by trypan blue staining (*n* = 4). (*C*) Time course experiments showing inhibition of *MALAT1* RNA expression (1 µM, *n* = 2, Optimization 4). EP(−) is non-oligo treatment, no electroporation control. All data are presented as ± STDEV.

We then moved from wild-type neuronal cells to FRDA patient-derived iPSC-NPCs and achieved good knockdown efficiencies and high cell survival (Fig. 4A,B). Efficient gene knockdown was observed from 1 to 4 d even though cells divided (and therefore diluted ASO) 2–3 times over that period (Fig. 4C). Gymnotic and lipid-mediated delivery were less potent, with gymnotic delivery yielding almost no inhibition when tested at 20 µM (Supplemental Fig. 2).

### Activation of *FXN* mRNA expression in FRDA iPSC-NPCs

**FIGURE 5.**
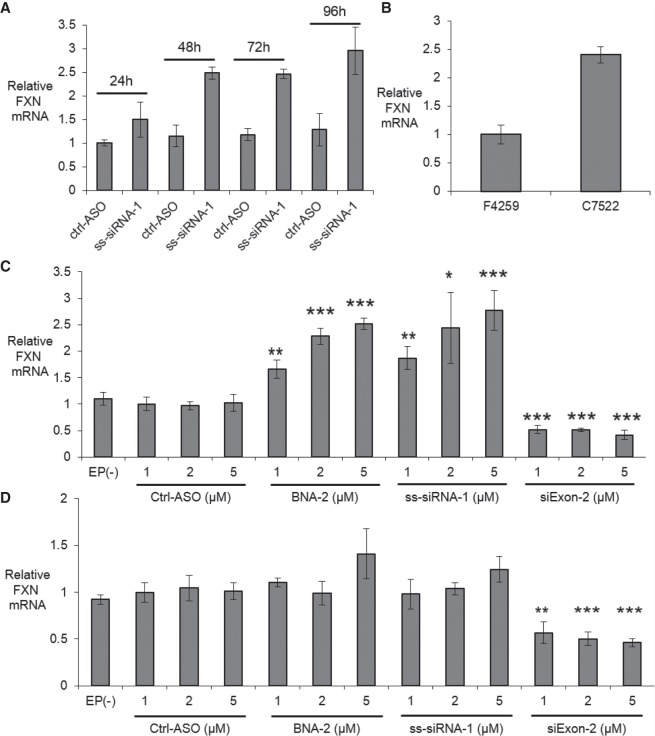
Activation of *FXN* expression and comparison to wild-type cells. (*A*) Time course showing activation of *FXN* mRNA expression starting 48 h post-transfection in FRDA patient-derived NPCs F4259 (5 µM, *n* = 2, Optimization 4). (*B*) Expression levels of *FXN* mRNA in FRDA patient-derived NPCs F4259 and wild-type NPCs C7522 (*n* = 4). (*C*) Activation in FRDA patient-derived NPCs F4259. (*D*) Effect of adding anti-AAG ASOs to wild-type NPCs C7522. Relative *FXN* mRNA levels were measured by RT-qPCR when transfected with oligonucleotides using Optimization 4 (*n* = 4) post 72 h transfection. EP(−) is non-oligo treatment, no electroporation control. All data are presented as ±STDEV. (*) *P* < 0.05, (**) *P* < 0.01, (***) *P* < 0.001, relative to EP (−) by Student *t*-test.

With a rapid and robust transfection method to neuronal cells in hand, we tested activation of *FXN* expression in FRDA iPSC-NPCs F4259. We prioritized testing ss-siRNA ss-siRNA-1 (Fig. 5A) due to its potent activation of *FXN* expression in fibroblasts. ss-siRNAs are single-stranded RNA compounds that contain chemical modifications that stabilize them while permitting action through the RNA interference (RNAi) pathway (Lima et al. 2012). We observed a 2.5-fold increase of FXN mRNA levels relative to the negative control ctrl-ASO and this increase was observed from 2 to 4 d post-transfection. For comparison, we measured levels of *FXN* RNA in wild-type C7522 iPSC-NPCs and observed a level 2.5 times higher than patient-derived F4259 cells (Fig. 5B). These data suggest that ASOs targeting the GAA repeat can restore *FXN* RNA expression to near normal levels in iPSC-NPCs.

We tested ss-siRNA-1 and another potent *FXN* activator, BNA-2, and tested the activation of *FXN* mRNA expression with three different concentrations (1, 2, and 5 µM). BNA-2 contains a 2′4′-constrained ethyl modification that promotes optimal binding affinity to complementary targets (Seth et al. 2008). All three concentrations of ss-siRNA-1 and BNA-2 activated *FXN* mRNA expression in a dose-dependent manner (Fig. 5C). As a positive control for transfection efficiency we used si-Exon2, a duplex RNA targeting the exon 2 region of *FXN* gene. Noncomplementary ss-siRNA ctrl-ASO did not activate FXN expression. Electroporation alone did not alter FXN mRNA expression (Supplemental Fig. 6).

We also tested activating oligonucleotides ss-siRNA-1 and BNA-2 in wild-type iPSC-NPC C7522 to test their effect in cells with normal *FXN* expression. These compounds did not significantly activate *FXN* mRNA expression in wild-type iPSC-NPCs (Fig. 5D), supporting the conclusion that activation in patient-derived cells was an on-target effect and that ASO activators cannot super-activate *FXN* expression above normal levels.

### Potency of gene activation: effect of mechanism and chemical modifications

**FIGURE 6.**
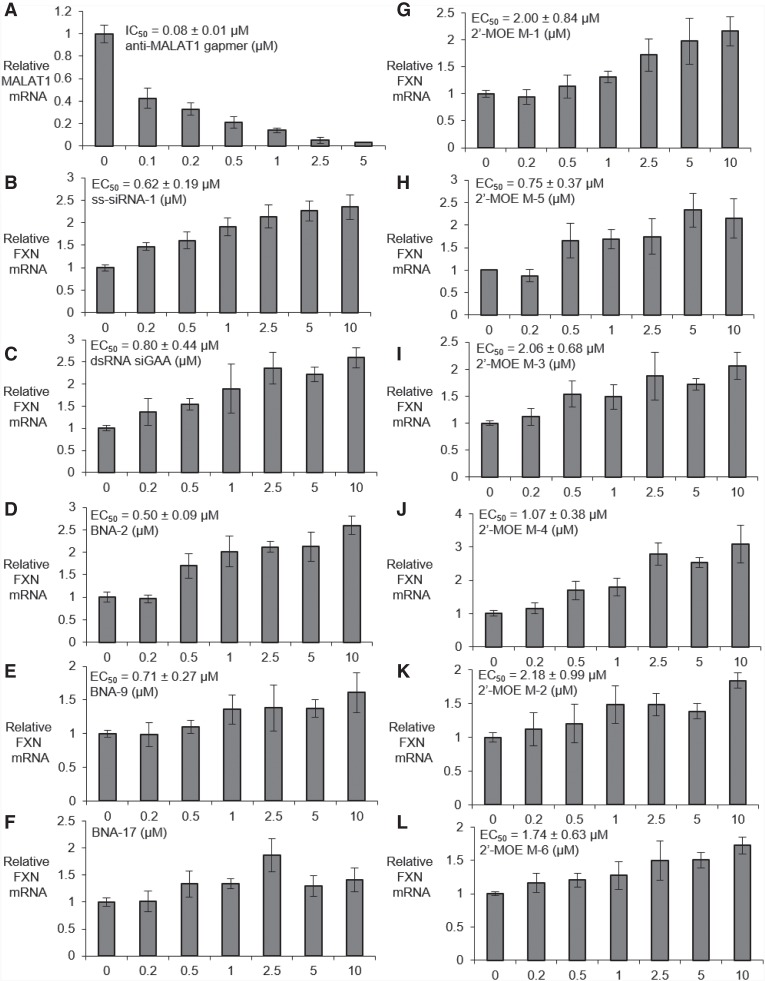
Dose dependent modulation of gene expression (Optimization 4) in FRDA patient-derived NPCs F4259 by (*A*) anti-MALAT1 (*n* = 6), (*B*) ss-siRNA-1, (*C*) Duplex RNA siGAA. (*D*–*F*) Dose-dependent activation of *FXN* mRNA by bridged nucleic acids (*D*) BNA-2, (*E*) BNA-9, and (*F*) BNA-17. (*G*–*L*) Dose-dependent activation of *FXN* mRNA by ASOs with 2′-O-MOE (*G*) M-1, (*H*) M-5, (*I*) M-3, (*J*) M-4, (*K*) M-2, and (*L*) M-6 (*n* = 4). All data are presented as ±STDEV.

We then performed dose response experiments to characterize the potency of oligonucleotides containing varied chemical modifications. Benchmark gapmer anti-MALAT1 possessed an IC_50_ value of 80 nM in FRDA iPSC-NPCs after 24 h of electroporation in FRDA iPSC-NPCs (Fig. 6A). The IC_50_ value of anti-MALAT1 was 190 nM after 72 h of electroporation in FRDA iPSC-NPCs (Supplemental Fig. 3).

For a full comparison of activity with nucleic acid design, we chose one duplex RNA, one ss-siRNA, three bridged nucleic acids and six ASOs with 2′-O-MOE modifications to test in FRDA iPSC-NPCs based on their potencies in FRDA patient-derived fibroblasts (Li et al. 2018; Shen et al. 2018). The EC_50_ values of the ss-siRNA ss-siRNA-1 and the duplex RNA siGAA were 620 and 800 nM (Fig. 6B,C). Constrained ethyl (cEt) modified ASO BNA-2 and BNA-9 showed EC_50_ values 500 and 710 nM, respectively. BNA-17 did not have a clear dose-response and was not scored as active (Fig. 6D–F).

For the six ASOs containing 2′-O-MOE, the EC_50_ value of the 16-mer ASO targeting GAA repeat sequence M-1 was 2 µM, while its 18-mer analog M-5 possessed a potency of 0.75 µM, suggesting longer sequences with 2′-O-MOE might increase the potency of *FXN* mRNA up-regulation (Fig. 6G,H). Similar trends were also observed in the two 2′-O-MOE ASOs M-3 and M-4 targeting GAA repeat register (EC_50_ 2.1 and 1.1 µM, respectively), and the two 2′-O-MOE ASOs M-2 and M-6 targeting AGA repeat register (EC_50_ 2.2 and 1.7 µM, respectively) (Fig. 6I–L).

### Activation of FXN protein expression

**FIGURE 7.**
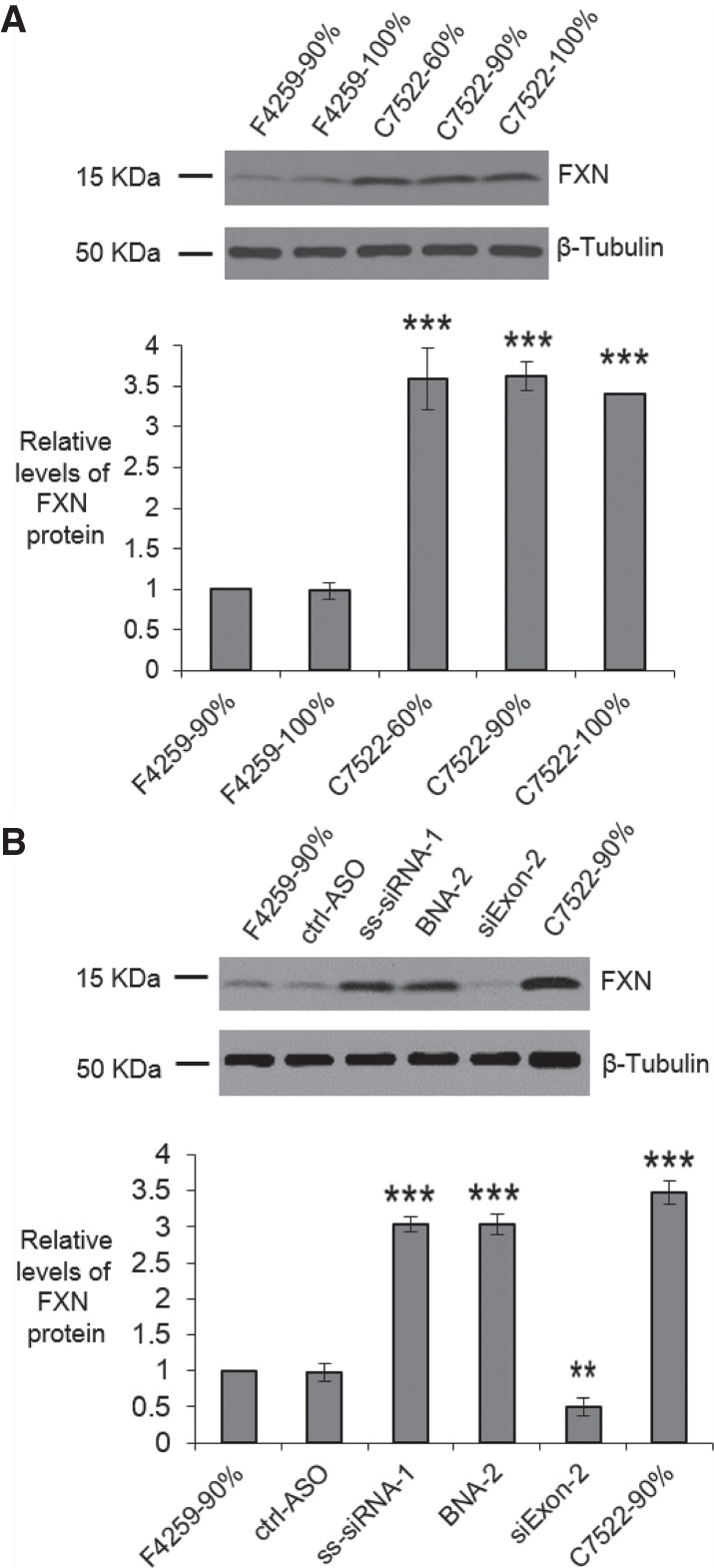
Activation of FXN protein expression, western analysis. (*A*) FXN protein in FRDA patient-derived NPCs F4259 and wild-type NPCs C7522 (*n* = 3) with different confluency. (*B*) FXN protein expression in FRDA patient-derived NPCs F4259 when transfected with oligonucleotides (5 µM) using Optimization 4 (*n* = 3) post 96 h transfection. All data are presented as ±STDEV. (*) *P* < 0.05, (**) *P* < 0.01, (***) *P* < 0.001, relative to F4259-90% by Student *t*-test.

Increased FXN protein expression will be necessary to treat FRDA and we chose ss-siRNA-1 and BNA-2 to test activation of FXN protein expression in FRDA iPSC-NPCs. For comparison, we first measured FXN protein in patient-derived and wild-type cells (Fig 7A). We examined cells cultured to different confluence to test if confluence affected protein expression. Consistent with our measurements of *FXN* mRNA, the FXN protein level in FRDA iPSC-NPCs F4259 is around 3.5-fold lower than that in wild-type iPSC-NPCs C7522.

We then examined the effect of electroporation of ss-siRNA-1 or BNA-2 on expression of FXN protein. We observed that either ss-siRNA-1 and BNA-2 at 5 µM increased FXN protein expression by threefold, close to the levels found in wild-type cells (Fig. 7B; Supplemental Fig. 5). This result is consistent with the activation of *FXN* mRNA expression by duplex RNA or ASOs and suggests that synthetic oligonucleotides can restore FXN protein expression to near normal levels in FRDA neuronal cells.

## DISCUSSION

### Cell culture models and synthetic oligonucleotides

Progress in the clinic has focused more attention on the development of synthetic nucleic acids as drugs. More physiologic disease targets are being considered and the standards for investing in a robust preclinical program are becoming more demanding. One way to build a case for vigorously pursuing a target is to conduct testing in cells that resemble the cell types that would be targets in patients. Such testing would assist the design of animal trials or might even substitute for animal testing if an adequate animal was unavailable.

### Electroporation: a robust tool for introducing synthetic nucleic acids into cells

iPS cells provide a source for differentiated cells that possess characteristics of target cells in vivo. Some differentiated cells cannot be transfected by standard lipid-mediated protocols or by gymnotic delivery, including the iPSC-NPCs used in this study. We have shown that the MaxCyte system and associated protocols provide an effective means to introduce ASOs and dsRNAs into iPSC-NPCs. We observed potent activities inside cell for gapmer-mediated inhibition of *MALAT1* gene expression and ASO or dsRNA-mediated activation of *FXN* expression.

Use of the MaxCyte system has several practical advantages. Electroporation was a fast and simple procedure. We had previously struggled with reproducibility when activating FXN expression in patient-derived fibroblast cells. The activation window between the induced and uninduced state was less than threefold. This relatively narrow window was often obscured by the variable efficiency of lipid-mediated transfection and toxicity associated with the transfection protocol. We found that electroporation was more robust. Toxicity was lower and successful experiments were a routine outcome achievable by novice experimenters. Our experience has demonstrated that electroporation can facilitate difficult experiments and permit rapid progress toward comprehensive compound testing. We note that other electroporation devices are also available and look forward to comparison testing and wider adoption of this class of instrumentation.

### Potential of synthetic oligonucleotides for activating *FXN* expression

Friedreich's ataxia has no curative treatments and the need for progress toward potential therapies is urgent. Moving our previously described nucleic acids directly to animal models would be an ideal next step and is being actively pursued. Friedreich's ataxia, however, is caused by an unusual mechanism that involves the mutant trinucleotide repeat forming an R-loop at the *FXN* locus. It is unclear how mouse models will mimic R-loop formation or the ability of a synthetic nucleic acid to block R-loop formation and activate gene expression. We chose, therefore, to investigate iPSC-NPCs because it offers a near-term test. Observation of gene activation would suggest further tests, while a failure to observed activation would call the hypothesis into question.

Our results demonstrate that dsRNAs and ASOs can activate FXN in the context of human iPSC-NPCs. The dsRNA and the ss-siRNA tested functions through the RNA interference mechanism. The ASOs function by sterically blocking the repeat. These findings suggest that either mechanism, RNAi or steric block, can be used for future development. We also observed that varied combinations of chemistry, including 2′-MOE, cEt, and 2′-F could be used in active ASO designs. These data reinforce the conclusion that there is substantial flexibility in future attempts to design activating compounds.

This good news for future development is balanced by results that highlight the need for improvement. Potencies of the best activating compounds were several fold lower than the potency for the benchmark ASO anti-MALAT-1 (Figs. 2, 6; Supplemental Fig. 4). While it is not possible to extrapolate with complete confidence between culture cells, animals, and human clinical trials, the result suggests that clinical development would benefit from the discovery of compounds that have potencies comparable to gapmer oligonucleotides that have already proven to be effective in humans.

### Future objectives

With this demonstration that synthetic oligonucleotides can activate FXN expression in neuronal cells, it is important to begin testing for activation in model mice possessing the human *FXN* gene containing the mutant expanded GAA repeats. Such studies should be designed to permit a direct comparison of FXN-activating ASOs with anti-MALAT1 or some other benchmark ASO. In parallel, continued effort should be spent optimizing ASOs for increased potency in neuronal cells.

### Conclusion

Our experiments have demonstrated that electroporation is a practical method for introducing duplex RNAs and ASOs into neuronal cells. Robust instrumentation and protocols allow rapid progress. We have used these protocols to demonstrate activation of *FXN* expression in iPSC-derived human neuronal cells. We achieved levels of activation that are like those observed in wild-type cells. Activation was a general property demonstrated by a diverse group of compounds. To continue to build a case for clinical development, animal testing and further optimization of potency are urgent goals.

## MATERIALS AND METHODS

### Tissue culture and transfection

Human motor neurons (iPSC-derived, normal) were purchased from iXCell Technologies (40HU-005) and were cultured in motor neuron maintenance medium (iXCell Technologies, MD-0022) at 37°C in 5% CO_2._ Two primary fibroblast lines derived from FRDA patient (F4259) and unaffected control individual (C7522) were reprogrammed to induced pluripotent stem cells (iPSCs) using integration-free Sendai virus transgene delivery (CytoTune 2.0 kit, ThermoFisher Scientific) according to the manufacturer instructions. Both iPSC lines were tested for pluripotency and differentiation capabilities (Li et al. 2015). Karyotype analysis, conducted at Cell Line Genetics, demonstrated a normal karyotype at the passage proceeding differentiation of the pluripotent cells to the NPCs (Supplemental Fig. 1). The iPSC lines were differentiated into neural progenitor cells (NPCs) via inhibiting TGF-β/SMAD signaling as described previously (Chambers et al. 2009). NPCs were maintained in STEMdiff neural progenitor medium (Stemcell Technologies). Cells were dissociated with 1× trypsin or StemPro Accutase Cell Dissociation Reagent (Gibco) + 10 µM Y27632 (Selleck Chemicals). Fibroblast cells, GM03816 (Coriell Institute, FRDA patient cell line) were cultured as described previously (Li et al. 2016).

MaxCyte system used preset protocols (Optimization 1 to 10, ranging from low energy to high energy) for most cell types. Transfection was performed by the MaxCyte STX scalable transfection system using Optimization 4 and Optimization 6 electroporation protocols with OC-100 cuvettes (MaxCyte, Inc.). Prior to electroporation, oligonucleotides or duplex RNAs were added to OC-100. Cells were thawed and added to the corresponding maintenance medium (10 mL), washed one time and resuspended in HyClone electroporation buffer (MaxCyte, Inc.). Cells were counted using trypan blue staining (TC20 Automated Cell Counter, Bio-Rad), 250,000 (HMNs) or 300,000–500,000 (neuronal progenitor cells) cells in the volume of 50 µL were added to OC-100 and electroporation was performed. Immediately after transfection, 50 µL of warm maintenance medium was added to the cuvettes, and the cuvettes were closed and rested in incubator (37°C and 5% CO_2_) for 15 min. Then, cells were plated (two wells per cuvette for RNA as two biological replicates, one well per cuvette for protein as one biological replicate) to 12-well or 24-well plates precoated with Corning Matrigel membrane matrix (Fisher Scientific, CB-40234). Lipid transfections were performed using Lipofectamine stem transfection reagent (Invitrogen) following reported protocols (Li et al. 2018; Shen et al. 2018). Gymnotic delivery (free uptake) was performed by mixing the oligonucleotides with cells and replacing fresh medium the next day. *FXN* and Metastasis Associated Lung Adenocarcinoma Transcript 1 (*MALAT1*) expression were assayed after 24–96 h by quantitative real-time PCR and western blot analysis. Cell viability was calculated by percentage of cell numbers at the time of harvest relative to cell numbers before transfection.

### Quantitative real-time PCR

Total RNA was harvested and treated with DNase (removing genomic DNA contamination) at 24–72 h post-transfection with NucleoSpin RNA XS kit (MACHEREY-NAGEL) following the manufacturer's recommended protocol. Equal amount of treated RNA (representing approximately the same number of cells and ranging from 0.2–1 µg of RNA) were reverse-transcribed using the High Capacity cDNA Reverse Transcription Kit (Applied Biosystems) and diluted to 60–100 µL final volume after reaction. Q RT-PCR was performed with two technical replicates per sample using iTaq Universal SYBR Green Supermix (Bio-Rad) with 5 µL of cDNA as template and primer pairs: FXN forward 5′-AAGCCATACACGTTTGAGGACTA-3′ and reverse 5′-TTGGCGTCTGCTTGTTGATCA-3′; HPRT forward 5′-AGTTCTGTGGCCATCTGC TTAGTAG-3′ and reverse 5′-AAACAACAATCCGCCCAAAGG-3′; MALAT1 forward 5′-CGGGTGTTGTAGGTTTCTCTT-3′ and reverse 5′-CCCACAAACTTGCCATCTACTA-3′; RPL19 forward 5′-AGCCTGTGACGGTCCATTCC-3′ and reverse 5′-CGGCGCAAAATCCTCATTCT-3′. Data were normalized relative to measured *HPRT* levels (*FXN*) or *RPL19* levels (*MALAT1*).

### Western blot analysis

Cell extracts were prepared using lysis buffer supplemented with 1% Protease Inhibitor Cocktail Set I (Calbiochem) as described previously (Watts et al. 2010; Smith et al. 2018). Proteins were separated on 4%–20% gradient Mini-PROTEAN TGXTM precast gels (Bio-Rad). After gel electrophoresis, proteins were wet transferred to nitrocellulose membrane (0.45 µm, GE Healthcare Life Sciences) at 100 V for 45 min (Zhang et al. 2018b). Membranes were blocked for 2 h at room temperature with 5% milk in 1× PBS containing 0.1% TWEEN-20 (PBST 0.1%). Blocked membranes were incubated with the primary antibodies at 4°C in PBST 0.1% with 1% milk on rocking platform overnight: anti-FXN at 1:20,000 (4F9, from Dr. Hélène Puccio at IGBMC, France) and anti-β-Tubulin at 1:5000 (Sigma-Aldrich, T5201). After primary antibody incubation, membranes were washed 4 × 10 min at room temperature with PBST 0.2% (1× PBS, 0.2% TWEEN-20) and then incubated for 1 h at room temperature with HRP-conjugated anti-Mouse IgG secondary antibody (Jackson ImmunoResearch, 715-035-150, FXN 1:20,000, β-Tubulin 1:10,000) in PBST 0.1%. Membranes were washed again 4 × 10 min in PBST 0.1% and 4 × 10 min in 1× PBS at room temperature. Washed membranes were soaked with HRP substrate according to manufacturer's recommendations (SuperSignal West Pico Plus Chemiluminescent substrate, Thermo Scientific) and exposed to films. The films were scanned and bands were quantified using ImageJ software.

### EC_50_/IC_50_ calculations

The program GraphPad Prism 7.03 was used to calculate EC_50_/IC_50_. The Hill equation (Goutelle et al. 2008) was used for fitting curves in the following form: Y = Y_0_ + (Y_max_ − Y_0_)X^n^/(K^n^ + X^n^), where Y is the normalized fold activation/inhibition, X is the oligo concentration, Y_0_ is baseline response (activation/inhibition at a oligo concentration 0), Y_max_ is the maximum fold activation/inhibition, K is the EC_50_/IC_50_ value and n is the Hill coefficient. Data sets from at least four replicates were used for curve fitting. The error of EC_50_/IC_50_ is standard error of the mean (SEM), which is calculated from combining the data of each individual dose curve.

## SUPPLEMENTAL MATERIAL

Supplemental material is available for this article.

## Supplementary Material

Supplemental Material
