# Supplementary material for: Efficient electroporation of neuronal cells using synthetic oligonucleotides: identifying duplex RNA and antisense oligonucleotide activators of human frataxin expression
Source: RNA. 2019 Sep;25(9):1118–29. doi: 10.1261/rna.071290.119 (PMC6800520; doi:10.1261/rna.071290.119)
Supplement: Supplemental Material [file supp_25_9_1118__index.html]

Efficient Electroporation of Neuronal Cells Using Synthetic Oligonucleotides: Identifying Duplex RNA and Antisense Oligonucleotide Activators of Human Frataxin Expression — Efficient Electroporation of Neuronal Cells Using Synthetic Oligonucleotides: Identifying Duplex RNA and Antisense Oligonucleotide Activators of Human Frataxin Expression — Efficient electroporation of neuronal cells using synthetic oligonucleotides: identifying duplex RNA and antisense oligonucleotide activators of human frataxin expression — Supplemental Material 

# Efficient electroporation of neuronal cells using synthetic oligonucleotides: identifying duplex RNA and antisense oligonucleotide activators of human frataxin expression

## Supplemental Material

- Supplemental\_Figures.pptx
