# Supplementary material for: Efficient electroporation of neuronal cells using synthetic oligonucleotides: identifying duplex RNA and antisense oligonucleotide activators of human frataxin expression
Source: RNA. 2019 Sep;25(9):1118–29. doi: 10.1261/rna.071290.119 (PMC6800520; doi:10.1261/rna.071290.119)
Supplement: Supplemental Material [file supp_071290.119_Supplemental_Figures.pptx]

## Slide 1
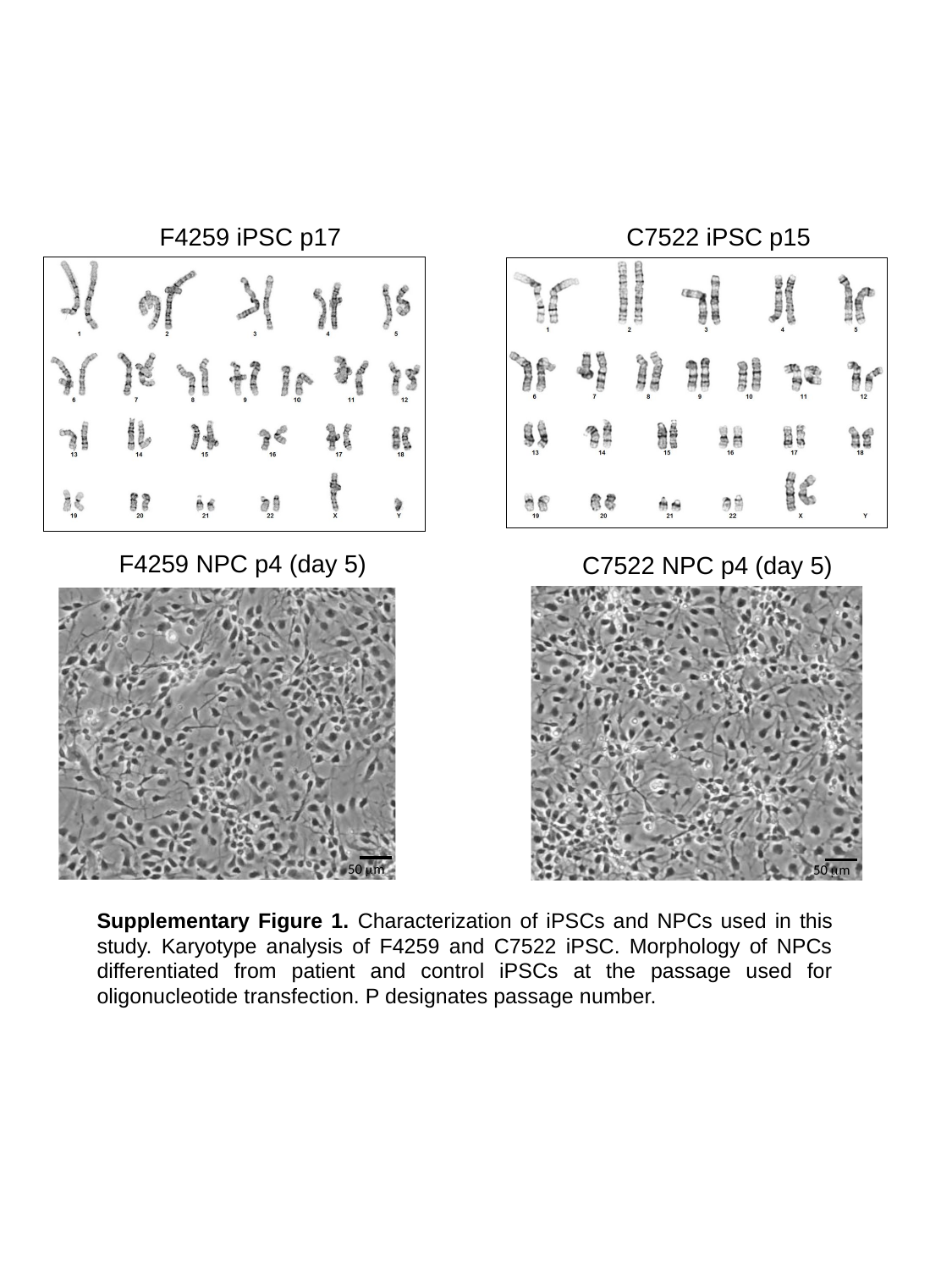

F4259 iPSC p17 C7522 iPSC p15
F4259 NPC p4 (day 5)
C7522 NPC p4 (day 5)
50 mm
50 mm
Supplementary Figure 1. Characterization of iPSCs and NPCs used in this study. Karyotype analysis of F4259 and C7522 iPSC. Morphology of NPCs differentiated from patient and control iPSCs at the passage used for oligonucleotide transfection. P designates passage number.

## Slide 2
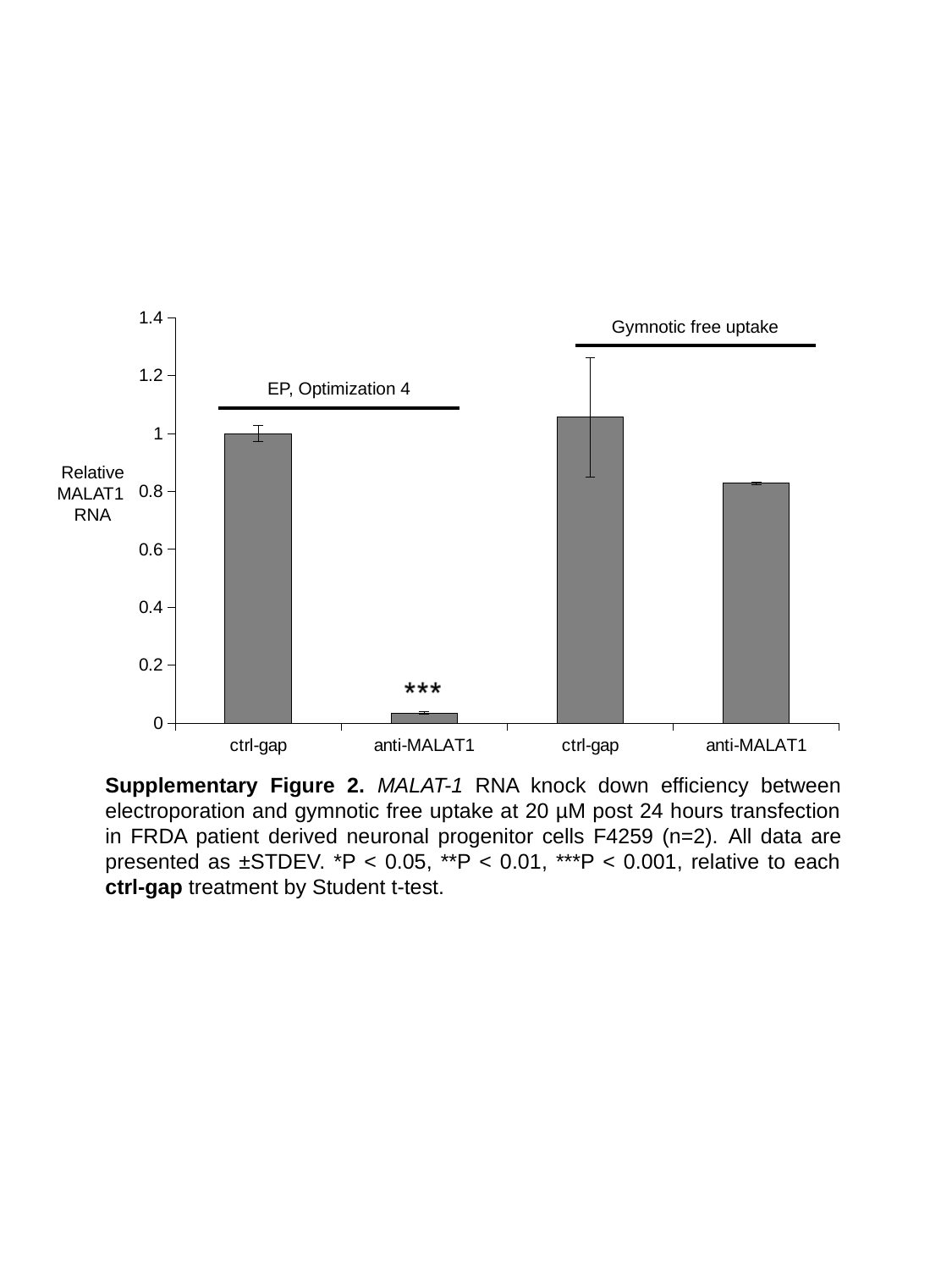

### Chart
| Category | |
|---|---|
| ctrl-gap | 1.0 |
| anti-MALAT1 | 0.0350148626642339 |
| ctrl-gap | 1.056606895301593 |
| anti-MALAT1 | 0.828592610982003 |Gymnotic free uptake
EP, Optimization 4
Relative
MALAT1
RNA
Supplementary Figure 2. MALAT-1 RNA knock down efficiency between electroporation and gymnotic free uptake at 20 µM post 24 hours transfection in FRDA patient derived neuronal progenitor cells F4259 (n=2). All data are presented as ±STDEV. *P < 0.05, **P < 0.01, ***P < 0.001, relative to each ctrl-gap treatment by Student t-test.

## Slide 3
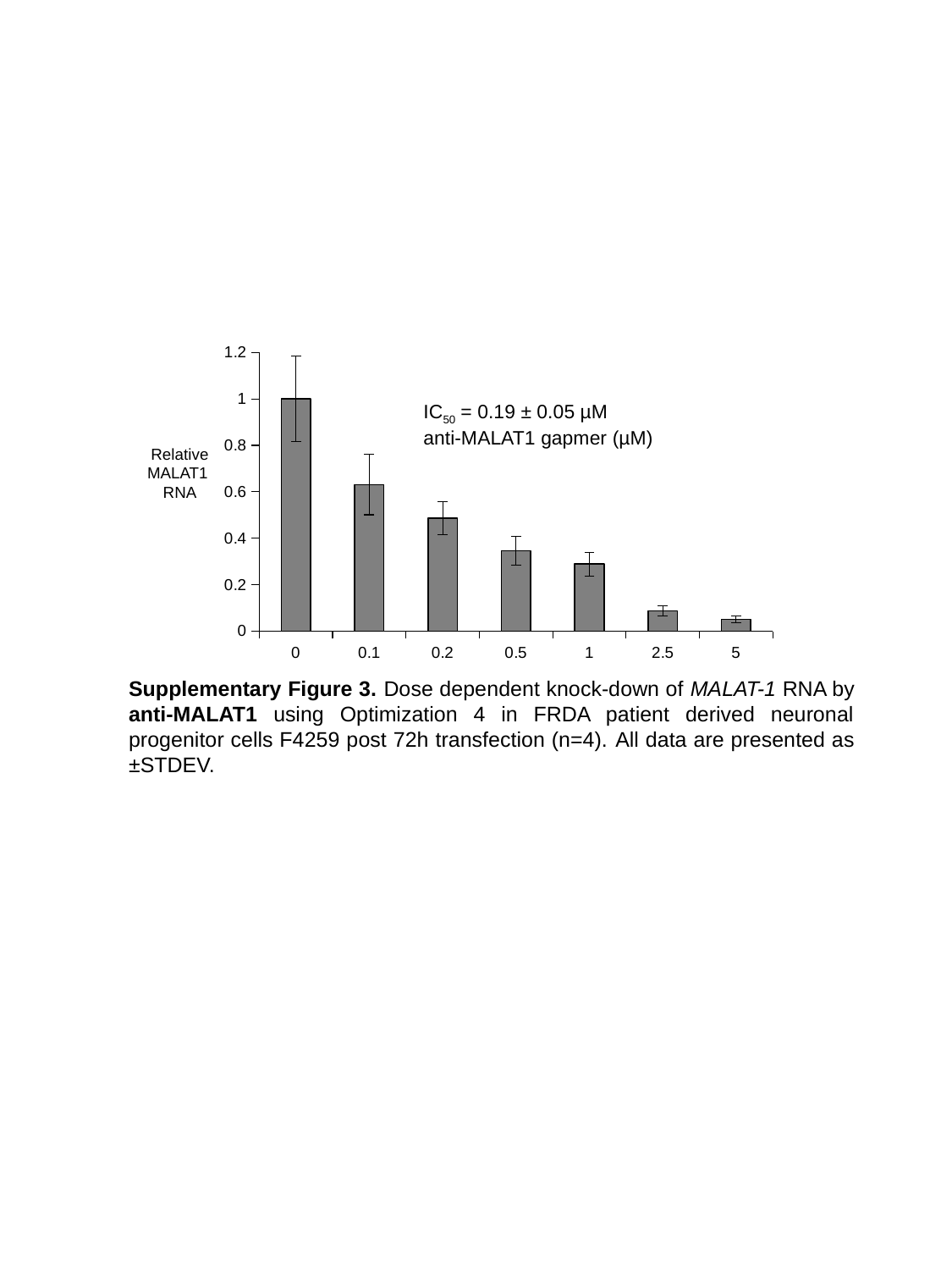

### Chart
| Category | |
|---|---|
| 0 | 1.0 |
| 0.1 | 0.6308532339019214 |
| 0.2 | 0.48594304940078326 |
| 0.5 | 0.3463822553550829 |
| 1 | 0.28962871697581666 |
| 2.5 | 0.08801107433507437 |
| 5 | 0.05196716521303467 |IC50 = 0.19 ± 0.05 µM
anti-MALAT1 gapmer (µM)
Relative
MALAT1
RNA
Supplementary Figure 3. Dose dependent knock-down of MALAT-1 RNA by anti-MALAT1 using Optimization 4 in FRDA patient derived neuronal progenitor cells F4259 post 72h transfection (n=4). All data are presented as ±STDEV.

## Slide 4
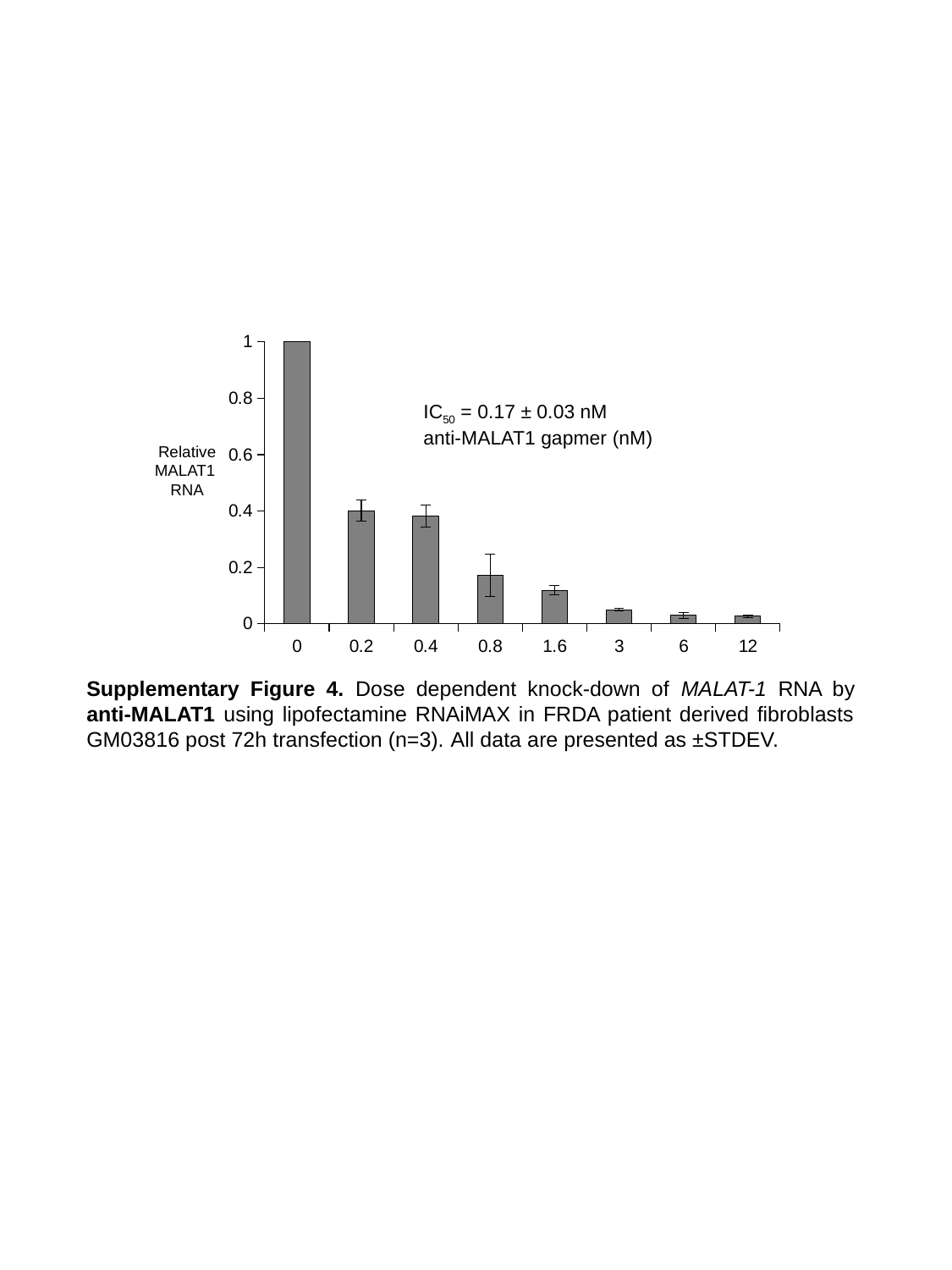

### Chart
| Category | |
|---|---|
| 0 | 1.0 |
| 0.2 | 0.4009572750282533 |
| 0.4 | 0.3821498904226818 |
| 0.8 | 0.17139237967464288 |
| 1.6 | 0.11932126378946076 |
| 3 | 0.04965635292524498 |
| 6 | 0.030523615267118484 |
| 12 | 0.027045791689908262 |IC50 = 0.17 ± 0.03 nM
anti-MALAT1 gapmer (nM)
Relative
MALAT1
RNA
Supplementary Figure 4. Dose dependent knock-down of MALAT-1 RNA by anti-MALAT1 using lipofectamine RNAiMAX in FRDA patient derived fibroblasts GM03816 post 72h transfection (n=3). All data are presented as ±STDEV.

## Slide 5
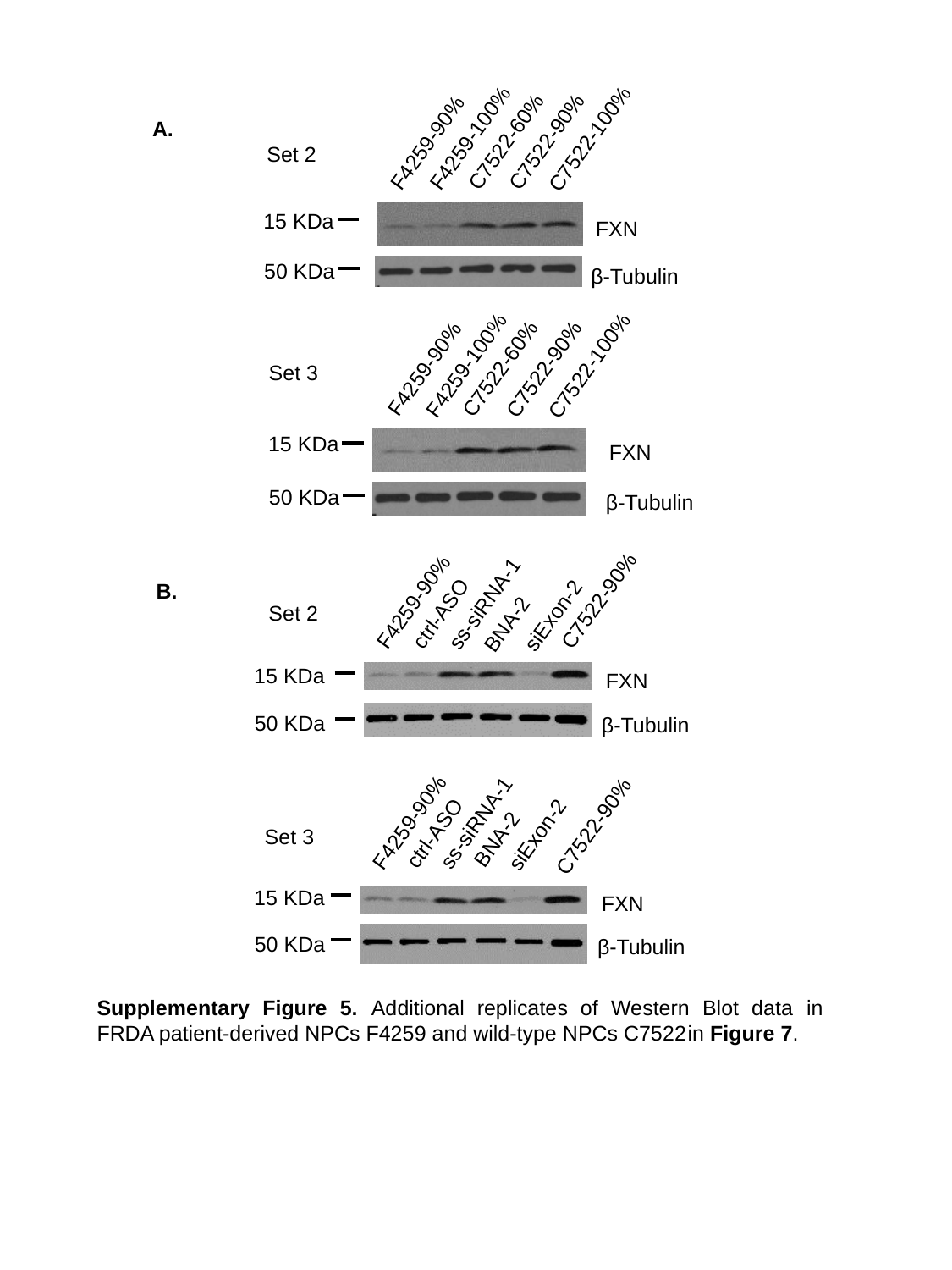

F4259-100%
C7522-100%
C7522-60%
C7522-90%
F4259-90%
Set 2
15 KDa
FXN
50 KDa
β-Tubulin
A.
F4259-100%
C7522-100%
C7522-60%
C7522-90%
F4259-90%
Set 3
15 KDa
FXN
50 KDa
β-Tubulin
C7522-90%
F4259-90%
ss-siRNA-1
Set 2
ctrl-ASO
siExon-2
BNA-2
15 KDa
FXN
50 KDa
β-Tubulin
B.
F4259-90%
ss-siRNA-1
C7522-90%
ctrl-ASO
siExon-2
Set 3
BNA-2
15 KDa
FXN
50 KDa
β-Tubulin
Supplementary Figure 5. Additional replicates of Western Blot data in FRDA patient-derived NPCs F4259 and wild-type NPCs C7522in Figure 7.

## Slide 6
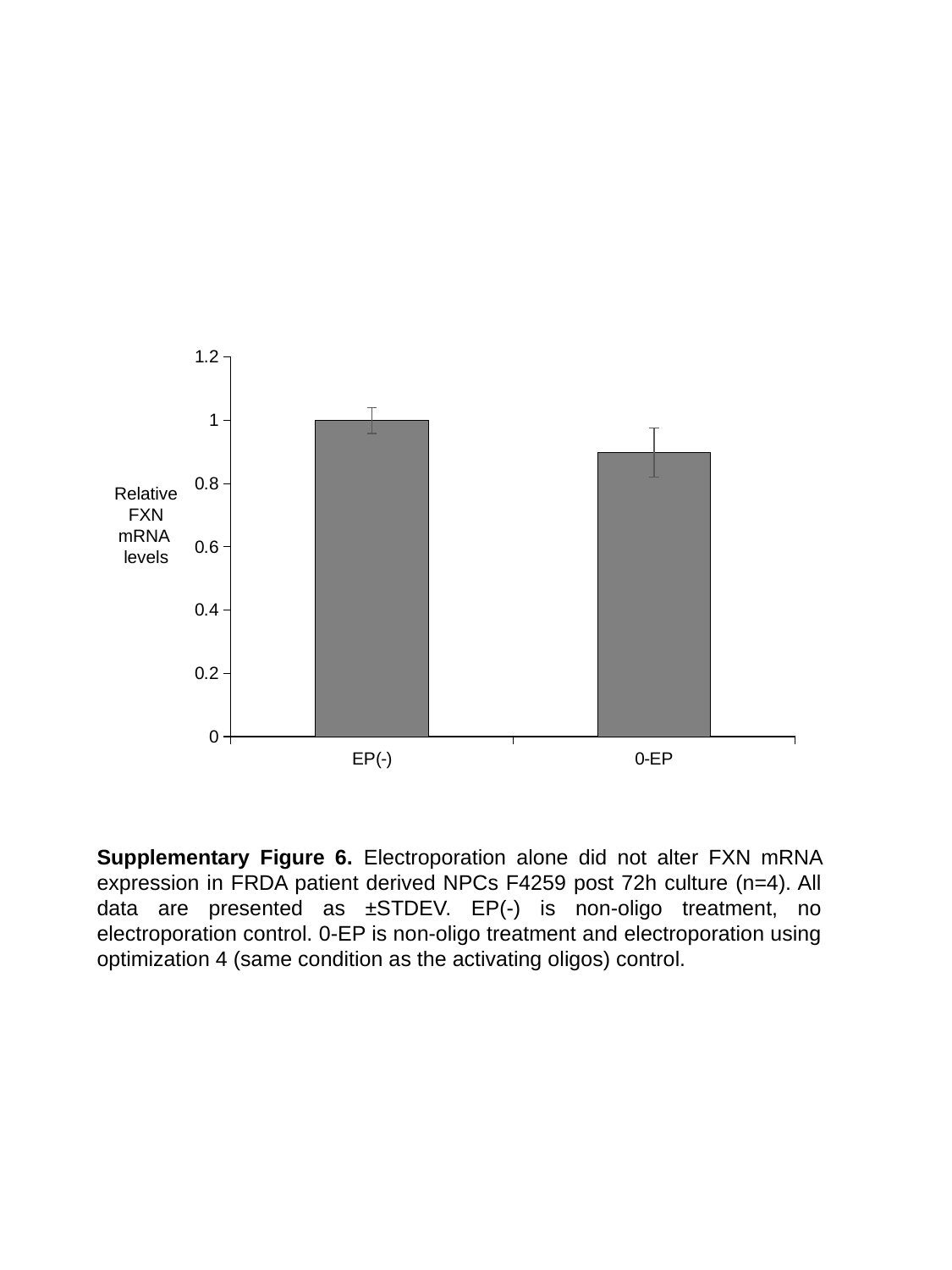

### Chart
| Category | |
|---|---|
| EP(-) | 1.0 |
| 0-EP | 0.8993659171222501 |Relative
FXN
mRNA
levels
Supplementary Figure 6. Electroporation alone did not alter FXN mRNA expression in FRDA patient derived NPCs F4259 post 72h culture (n=4). All data are presented as ±STDEV. EP(-) is non-oligo treatment, no electroporation control. 0-EP is non-oligo treatment and electroporation using optimization 4 (same condition as the activating oligos) control.
